# Simulating the bounds of plausibility: Estimating the impact of high-risk versus population-based approaches to prevent firearm injury

**DOI:** 10.1371/journal.pone.0269372

**Published:** 2022-06-02

**Authors:** Katherine M. Keyes, Ava Hamilton, Melissa Tracy, Rose M. C. Kagawa, Veronica A. Pear, David Fink, Charles C. Branas, Magdalena Cerdá

**Affiliations:** 1 Department of Epidemiology, Mailman School of Public Health, Columbia University, New York, New York, United States of America; 2 Department of Epidemiology and Biostatistics, State University of New York at Albany, Albany, New York, United States of America; 3 Department of Emergency Medicine, Violence Prevention Research Program, School of Medicine, University of California, Davis, Sacramento, California, United States of America; 4 Division of Translational Epidemiology, New York State Psychiatric Institute, New York, New York, United States of America; 5 Department of Population Health, New York University Langone Health, New York, New York, United States of America; Shandong University of Science and Technology, CHINA

## Abstract

**Background:**

Firearm violence remains a persistent public health threat. Comparing the impact of targeted high-risk versus population-based approaches to prevention may point to efficient and efficacious interventions. We used agent-based modeling to conduct a hypothetical experiment contrasting the impact of high-risk (disqualification) and population-based (price increase) approaches on firearm homicide in New York City (NYC).

**Methods:**

We simulated 800,000 agents reflecting a 15% sample of the adult population of NYC. Three groups were considered and disqualified from all firearm ownership for five years, grouped based on prevalence: low prevalence (psychiatric hospitalization, alcohol-related misdemeanor and felony convictions, 0.23%); moderate prevalence (drug misdemeanor convictions, domestic violence restraining orders, 1.03%); and high prevalence (all other felony/misdemeanor convictions, 2.30%). Population-level firearm ownership was impacted by increasing the price of firearms, assuming 1% price elasticity.

**Results:**

In this hypothetical scenario, to reduce firearm homicide by 5% in NYC, 25% of the moderate prevalence group, or 12% of the high prevalence group needed to be effectively disqualified; even when all of the low prevalence group was disqualified, homicide did not decrease by 5%. An 18% increase in price similarly reduced firearm homicide by 5.37% (95% CI 4.43–6.31%). Firearm homicide declined monotonically as the proportion of disqualified individuals increased and/or price increased. A combined intervention that both increased price and effectively disqualified “high-risk” groups achieved approximately double the reduction in homicide as any one intervention alone. Increasing illegal firearm ownership by 20%, a hypothetical response to price increases, did not meaningfully change results.

**Conclusion:**

A key takeaway of our study is that adopting high-risk versus population-based approaches should not be an “either-or” question. When individual risk is variable and diffuse in the population, “high-risk approaches” to firearm violence need to focus on relatively prevalent groups and be highly efficacious in disarming people at elevated risk to achieve meaningful reductions in firearm homicide, though countering issues of social justice and stigma should be carefully considered. Similar reductions can be achieved with population-based approaches, such as price increases, albeit with fewer such countering issues.

## Introduction

In 2019, approximately 40,000 individuals were killed with firearms in the United States (US); among those, almost 37% were designated as homicides [1]. Mass shootings regularly capture national headlines and media attention [2], often coupled with calls for increased governmental regulation of firearm purchases, ownership, and use [3]. However, mass shootings are by far the smallest category of gun death. Further, there are over two to three times as many non-fatal firearm injuries as death [4,5], which places an extraordinary burden on emergency departments and trauma centers throughout the country.

Leading prevention strategies prohibit firearm access based on individual characteristics thought to increase the risk of firearm injuries, such as mental health ajudications and criminal records. Such strategies are “high-risk approaches” to intervention [6], in that individuals belonging to specific groups thought to be at elevated risk for gun violence are specifically targeted [7,8]. These approaches aim to reduce risk among high-risk individuals, particularly in the near term. However, the true risk associated with prohibiting categories varies greatly [9], with some actually at relatively low risk of violence perpetration. While high-risk approaches such as disqualifications are porous in effectivness if not enforced or not well targeted based on risk, they remain a central yet controversial focus of firearm control in the US [10], and are thus important to consider. A high-risk approach may even have population-level impacts when the predictive power is strong, prevalence is high, and the response mechanism is effective and has sufficient reach [11,12]. However, it is challenging for high-risk approaches to produce population-level effects given the ubiquity of gun violence, the difficulty of predicting who will commit harms with firearms [13], possible misclassification of at-risk individuals, potential loopholes in the background check system, and the existence of the black market [14–17].

As the US is estimated to have the highest rates of civilian firearm ownership in the world [18], an alternative to the high-risk approach would be to follow the examples of other countries (e.g., Australia [19]) and limit access to firearms across the population, regardless of risk, a “population-based approach” [6]. Population-based methods that are highly effective typically involve product price and use. Taxes on products that adversely impact public health are among the most effective [20–25] and cost-effective [22,26–28] means of reducing negative consequences of use, both in the US and worldwide. While there are many population-based approaches to firearm control that could hypothetically be considered to reduce firearm violence in the US, there are increasing efforts to influence firearm pricing through taxation [29], and implementation of ammunition and firearm purchase taxes, thus a focus on violence prevention through price control in the US is a feasible consideration.

The ability of taxation to influence product sales depends on the product’s price elasticity. Price elasticity refers to the percentage change of a product in response to a percentage change in another, such that if a product’s price elasticity of demand is -1.5, a 10% increase in price causes the quantity demand to fall 15%. Available evidence indicates that firearms used for criminal purposes have a relatively low elasticity [30–32], but firearms and ammunition purchased for recreation and safety are relatively price elastic [33,34]. Cook et al. demonstrated that homicide is elastic to gun ownership prevalence, suggesting that measures to reduce ownership, including gun costs, are viable approaches to reduce homicide but may have limited impact on other crimes [35]. While firearms used for assaults resulting in homicide are often carried by individuals who procure them illegally [36], most firearms in the US are obtained through legal means [37], either through a licensed dealer or secondary market [38]. Thus, there is potential to affect public health outcomes by controlling the legal market.

Evaluating the potential impact of high-risk versus population-based approaches to reducing firearm violence ideally would involve randomized trials or other experimental evidence [39,40], which are not easy to conduct. Instead, we can use simulation methods to illustrate the principles that underlie high-risk and population-based approaches, alone and in combination, under different assumptions [40–42]. Such hypothetical scenarios have the unique advantage of allowing us to quickly illustrate numerous interventions, vary assumptions about their enforcement, and leverage available empirical information in the literature regarding firearm injury and purchase denial criteria. However, simulation scenarios are imperfect. It can be challenging to estimate the effects of gun disqualifications as there are many groups already disqualified who have used firearms (e.g., based on age, immigration status, involvement with the legal system) and limited evidence on price elasticity across levels of firearm ownership, especially given other data limitations.

We illustrate central principles of the high-risk versus population-based approach using an agent-based modeling simulation to explore how firearm disqualifications and price elasticity affect firearm ownership and use in violence in synthetic New York City. The scenarios are hypothetical abstractions of the ‘real world,’ given data limitations and the need to make varying assumptions about the proportion of owned guns used in violence. Nevertheless, theoretical examples demonstrate public health approaches to reducing firearm-related morbidity and mortality through simulating health outcomes given changes in access to firearms.

In sum, to reduce firearm-related homicide in the population by increasingly high percentages, we tested adjusting the efficacy of disqualification criteria and increasing firearm and ammunition pricing. Further, we estimated the combined effect of disqualification criteria and price increases on firearm-related homicide. In doing so, we present the potential bounds of reductions in firearm-related homicide for each approach.

## Methods

We developed an agent-based model (ABM) simulating the dynamic processes contributing to firearm-related homicide in New York City (NYC). Fig A.1.1 in S1 Appendix illustrates the relations included in the model [42–45]. In addition, S1 Appendix includes a description of the model following the ODD (Overview, Design concepts, Details) protocol [46,47], initialization parameters and default values (Table A1.2 in S2 Appendix), flow charts illustrating steps in the model (Figs A1.2 and A1.3 in S1 Appendix), and final calibration formulae for key model parameters. The data used to parameterize this model consists of previously collected, de-identified, aggregated data. The study meets the requirements by Columbia University’s Institutional Review Board (#AAAO6752) for a waiver of informed consent under 45 CFR 46.116(a) and 45 CFR 46.116(d).

### Agent population and neighborhoods

The population of 800,000 agents was initialized to approximate a 15% sample of the NYC adult population aged 18–84 years in 2010 [42,48–51]. Agent attributes are described fully in S1 Appendix. Agents were assigned to neighborhoods, proportionate to population and size; distributions of age, sex, race/ethnicity, and household income matched Census data for the 59 NYC community districts in 2010 [49]. New agents enter the model at age 18 and age through the lifecourse; their behaviors and outcomes are influenced by past trends as they enter the model.

### Social network and neighborhood influences

Each agent was assigned a target number of close social ties [52] based on age, sex, race/ethnicity, education, gun-carrying and ownership, drinking, drug use, and spatial proximity [52,53]. Agents’ probability of involvement in violence or gun-carrying increased if they had a social tie involved with violence or gun-carrying [54]. As a result, homicides and firearm-related homicides were clustered in certain social networks, following observed real-life dynamics.

Agents were embedded in neighborhoods. Predictive equations drawing on data from NYC were used to model the strength of the relationship between neighborhood characteristics (e.g., demographics, average mental health and suicide, violence and substance use) and homicide (S1 Appendix).

### Aging, mortality, movement

Each model step represented one year, and the model ran for 30 years. At each time step, agents aged, a proportion of agents moved to a new neighborhood [55,56], and agents died consistent with 2010 NYC adult all-cause mortality [57].

### Gun-carrying and ownership

At each timestep, agents could have firearms in their household and separately carry firearms. We modeled household guns and gun-carrying independently in order to allow agents to use guns without legal purchase (e.g., through non-licensed or illegal markets or exchanged within their social network). Probabilities of household firearms and gun-carrying were calibrated to National Comorbidity Survey Replication (NCS-R) [58] estimates based on demographics, history of victimization and perpetration, and substance use. Previous lifetime ownership influenced ownership at each timestep based on data from a survey conducted among US adults 18 years or older in 2013 by (http://www.yougov.com) [59]. We also allowed for agents with social network ties to agents who carried or had household firearms to use those firearms (S1 Appendix) [54].

### High-risk approaches to firearm ownership disqualification

We chose firearm disqualification categories based on their similarity with prohibited groups identified in the Gun Control Act of 1968 or state policies limiting firearm access by group with some variation in definitions based on data availability or for the purpose of exploration. We grouped examples of firearm disqualifications based on the prevalence of the criteria in the population to demonstrate how the size of the group targeted for disqualification is related to population impact [60,61]; membership in each group was not mutually exclusive. Details on data used for each category are provided in sections below.

*Low prevalence disqualifications* included 1) Office of Mental Health (OMH)-identified psychiatric hospitalization (throughout the model, the yearly average number of hospitalizations was 112/100,000), 2) alcohol-related misdemeanor (117/100,000), and 3) and felony (10/100,000) convictions; the rate of firearm-related homicide among the low prevalence group was 12/100,000. We note that psychiatric hospitalization alone does not result in firearm prohibition in much of the US. However, we use it in our models as a proxy for the prohibiting category, “committed to a mental institution”, which often does not include voluntary admissions. The association between mental illness and firearm violence is small and inconsistent [62,63], whereas alcohol-related convictions substantially increase risk of firearm violence [64].

*Moderate prevalence disqualifications* included 1) drug misdemeanor convictions (532/100,000) and 2) DVROs (517/100,000); the rate of firearm-related homicide among the moderate prevalence group was 22/100,000 in the model. Drugs convictions [65,66] and intimate partner violence [67] are both substantially associated with an increased risk of firearm violence.

*High prevalence disqualifications* included 1) all other felony convictions (i.e., drug and violent felonies) (753/100,000) and 2) misdemeanor convictions (i.e., larceny, impersonation) (1,652/100,000); the rate of firearm-related homicide among the high prevalence group was 17/100,000. People convicted or arrested for a violent felony or misdemeanor [68] are at a substantially increased risk for firearm violence. Less is known about associations with nonviolent misdemeanor convictions. Agents who met multiple groups’ criteria were disqualified from gun ownership based on the criteria met for their first disqualification. Agents meeting criteria for each prohibition were restricted from gun ownership and purchasing for five years. This disqualification timeframe is recommended for mental health hospitalization based on the Consortium for Risk-Based Firearm Policy [60], and for consistency, we set all interventions to five years. Gun-carrying remained possible for prohibited agents, as outlined below.

### Population approach to firearm ownership: Price increases

We simulated the impact of increasing price on firearm ownership by implementing price elasticity based on empirical evidence [31,38]. While information on the specific price elasticity of firearms in the US is scant, prior studies have shown that legal handgun and other gun purchases are sensitive to price [31,38]. Ownership was determined based on increasing the price by a certain percentage, multiplied by the price elasticity. Note that while we conceptualize this as a price increase through taxation, the model is agnostic to the mechanism through which the price was increased. Thus, this simulation is conceptualized as an intervention that effectively increases the cost of gun ownership through taxation, ammunition taxes, or other interventions that would manipulate the price of legal market firearms. Elasticity was varied in sensitivity analysis. Gun carrying did not increase due to changes in illicit ownership after price increases in the base model; rather the increase in illicit gun carrying after price increases was modeled in a sensitivity analysis (see below).

### Psychiatric hospitalization

We modeled psychiatric hospitalization using data from the 2013 Patient Characteristics Survey. This questionnaire collects information on all individuals in the public mental health system in New York, including inpatient psychiatric stays [69,70]. We calibrated the number of psychiatric hospitalizations to match the number of NYC adults hospitalized for psychiatric reasons identified by the NY State Office of Mental Health, based on race, age, and borough (i.e., Bronx, Brooklyn, Manhattan, Queens, Staten Island).

### Perpetration, arrests, convictions, and incarceration

Agents could be arrested, charged, and convicted with a felony or misdemeanor. Arrest probabilities were calculated from 2008–2014 National Survey on Drug Use and Health (NSDUH) data and included past 12-month arrests and reason for arrest (see S1 Appendix for equations).

After an alcohol-related, drug-related, or other type of arrest, agents were convicted of a misdemeanor or felony. However, under New York Penal Law §70.02, any crime designated as violent is considered a felony. We calculated conviction probabilities from the New York State Division of Criminal Justice Services (DCJS) 2011–2014 data for NYC based on sex, race, age, and borough. At each step, an agent could have up to seven convictions: a violent felony, an alcohol-related misdemeanor or felony, a drug-related misdemeanor or felony, or another type of misdemeanor or felony.

An agent convicted of a felony had a probability of incarceration based on NYC Department of Corrections [71], Justice Atlas data [72], and Survey of Inmates in State and Federal Correctional Facilities (SISCF) data [73]. Details on agent activity while incarcerated can be found in S1 Appendix.

### Intimate partner violence and domestic violence restraining orders

Agents could be victims and perpetrators of intimate partner violence (IPV). Using NCS-R data, probabilities were based on demographics, relationship and cohabitation status, household firearms and carrying status at previous time steps, history of violent and intimate partner victimization and perpetration, and substance use. Rates were calibrated to National Intimate Partner and Sexual Violence Survey 2010 report data [74]. IPV perpetrators could also have a Domestic Violence Restraining Order (DVRO) based on New York State Unified Court System [75].

### Homicide outcomes

Potential victims and perpetrators of violence were identified at each timestep, and violent incidents occurred, including homicides. Potential perpetrators (i.e., agents with a high predicted probability of perpetrating violence) searched a 15-cell radius around their location for potential victims (i.e., agents with a high probability of being victimized); any such agents who had not already been victimized at that time step were matched to a perpetrator. The potential victim was protected from violence only if a police officer was present within a 2-cell radius of the victim. Perpetration-victim matches could be due to IPV, which was modeled probabilistically based on sex and marital/dating status.

If the assault victim (IPV or non-IPV) was also a potential homicide victim, based on Office of Chief Medical Examiner (OCME) data [76], the violent incident became a homicide. Rates were calibrated to 2008–2014 NYC vital statistics data [77] based on race, sex, age, drug and alcohol use [78,79].

Homicide outcomes were influenced by social network [54,80], incarceration [81], and firearm ownership statuses [82,83]. Additionally, firearm-related homicide probability increased due to firearm ownership [83,84] and carrying statuses [82]. When a homicide occurred, it was determined to be firearm-related if the victimized agent was a potential firearm-related homicide victim and the victim or perpetrator owned, carried, or had access to a firearm through their social network.

### Model calibration and intervention scenarios

During model calibration, ABM estimates were compared to empirical data. An iterative process [85] was used to adjust predictive equations and initial conditions in the model (see Table 1).

**Table 1:** Estimates of firearm related homicide, low, moderate, and high prevelance risk groups, and firearm carrying and ownership from the ABM and empirical data sources in NYC when available, and from the National Comorbidity Survey Replication.

	ABM Estimate rate/100,000 (95% C.I.)^a^	Empirical estimates
Annual firearm related homicide	4.04 (3.7, 4.47)	3.96^b^
**Low Prevalence**		
OMH-identified psychiatric hospitalization	112.4 (109.6, 114.8)	108.02^c^
Alcohol misdemeanor conviction	117.2 (114.0, 119.3)	116.06^d^
Alcohol felony conviction	9.8 (9.2, 10.5)	9.95^d^
**Moderate Prevalence**		
Drug misdemeanor conviction	531.6 (525.1, 538.9)	525.74^d^
DVRO last year	517.3 (511.7, 521.8)	505.10^d^
**High Prevalence**		
Felony conviction	753.2 (745.0, 762.5)	758.05^d^
Misdemeanor Conviction	1,652 (1638, 1665)	1675.55^d^
Firearm ownership (%)	21.9 (21.8, 22.0)	22.03^e^
Firearm carrying status (%)	4.00 (3.99, 4.03)	3.94^e^

a. Median and 95% CI from 50 runs of ABM.

b. CDC WONDER (2008–2014). Underlying Cause of Death.

c. Treatment from NY State Office of Mental Health.

d. New York State Division of Criminal Justice Services (2011–2014). Dispositions.

e. National Comorbidity Survey Replication (NCS-R, 2001–2003), weighted to 2010 demographics.

After a burn-in period to stabilize estimates, each model scenario was run 100 times for 30 years. The mean across runs was reported for outcomes of interest; 95% confidence intervals (CI) reflect variation across runs. The model was developed using the Recursive Porous Agent Simulation Toolkit for Java (RepastJ, version 3.0) and implemented in Eclipse (version 4.6.1).

For the high-risk intervention approach, we determined what proportion of individuals (within each prevalence category) would need to be effectively disarmed for the homicide rate to decrease by ~5%. We then re-estimated how effective firearm disqualification criteria would need to be across prohibited groups to obtain higher reductions in homicide, up to a 100% effectiveness assumption. Similarly, for the population approach, we adjusted the increase in price necessary to reduce firearm-related homicide by 5%, 10%, etc. Because of the stochastic nature of the agent-based model, there was random variation in the percent reductions across simulations that approached the target decrease in firearm-associated homicide.

Finally, we estimated the combined impact of high-risk and population approaches. For each reduction bracket (e.g., 5%, 10%, etc.), we combined the estimates of the proportion of the prohibited group effectively prevented from ownership along with the needed price increase to estimate the decrease in firearm-related homicide if all strategies were implemented simultaneously. For example, if 25% of the moderate prevalence prohibited group needs to be effectively disqualified *or* price needs to increase by 18% to achieve 5% reduction in homicide, the combined intervention effect estimates the expected reduction in homicide with 25% disqualification in the moderate prevalence prohibited group *and* 18% price increase.

### Sensitivity analyses

1) Because data on the price elasticity of firearms is relatively scarce, we varied the price elasticity to both 0.5% and 2%. 2) Given the realistic assumption that some people will purchase a gun illegally (via a straw purchase, borrowing, or theft) when priced out of the legal market [10,86], we increased gun-carrying—our marker of unlawful access to firearms—(by 5%, 10%, and 20%) when the price increased in a sensitivity analysis. Specifically, for each 1% increase in price, we increased the prevalence of gun carrying (regardless of gun ownership) by 5, 10, and 20%.

## Results

Table 2 displays the simulation results of reducing firearm-related homicide in 5% brackets, up to 25%. We show the percentage of individuals who must effectively be disarmed (percent efficacy) in each prohibited group and the firearm price increase required to achieve the percentage reduction.

**Table 2:** Simulation of gun-related homicide in New York City after implementing firearm disqualification and price increases with a 1% price elasticity.

Intervention	Percent Efficacy	Individual Intervention Effects^a^				Combined Intervention Effects^b^			
		Firearm-Related Homicide Rate/100,000		% Decrease in Firearm-Related Homicide		Firearm-Related Homicide Rate/100,000		% Decrease in Firearm-Related Homicide	
		Mean	(95% CI)	Mean	(95% CI)	Mean	(95% CI)	Mean	(95% CI)
**Baseline**		4.04	(4.00, 4.08)			4.04	(4.00, 4.08)		
**Target: 5% Decrease in Homicide**									
** Low prevalence groups**	100% *	3.96	(3.92, 4.00)	1.96	(1.07, 2.85)	3.54	(3.50, 3.58)	12.34	(11.40, 13.27)
** Moderate prevalence group**	25% *	3.83	(3.80, 3.86)	5.15	(4.35, 5.95)				
** High prevalence groups**	12% *	3.82	(3.79, 3.86)	5.33	(4.45, 6.20)				
** Increase in price**	18% †	3.82	(3.78, 3.86)	5.37	(4.43, 6.31)				
**Target: 10% Decrease in Homicide**									
** Low prevalence groups**	100% *	3.96	(3.92, 4.00)	1.96	(1.07, 2.85)	3.18	(3.15, 3.22)	21.20	(20.35, 22.05)
** Moderate prevalence group**	55% *	3.64	(3.61, 3.66)	9.99	(9.37, 10.62)				
** High prevalence groups**	25% *	3.63	(3.59, 3.66)	10.23	(9.39, 11.08)				
** Increase in price**	35% †	3.65	(3.61, 3.68)	9.65	(8.78, 10.52)				
**Target: 15% Decrease in Homicide**									
** Low prevalence groups**	100% *	3.96	(3.92, 4.00)	1.96	(1.07, 2.85)	2.81	(2.78, 2.84)	30.32	(29.58, 31.06)
** Moderate prevalence group**	100% *	3.39	(3.35, 3.43)	16.07	(15.15, 16.98)				
** High prevalence groups**	40% *	3.42	(3.39, 3.45)	15.29	(14.48, 16.11)				
** Increase in price**	70% †	3.42	(3.38, 3.45)	15.41	(14.52, 16.31)				
**Target: 20% Decrease in Homicide**									
** Low prevalence groups**	100% *	3.96	(3.92, 4.00)	1.96	(1.07, 2.85)	2.65	(2.62, 2.68)	34.43	(33.64, 35.21)
** Moderate prevalence risk group**	100% *	3.39	(3.35, 3.43)	16.07	(15.15, 16.98)				
** High prevalence groups**	60% *	3.21	(3.18, 3.24)	20.55	(19.78, 21.32)				
** Increase in price**	110% †	3.22	(3.18, 3.25)	20.37	(19.52, 21.23)				
**Target: 25% Decrease in Homicide**									
** Low prevalence groups**	100% *	3.96	(3.92, 4.00)	1.96	(1.07, 2.85)	2.45	(2.42, 2.48)	39.40	(38.66, 40.14)
** Moderate prevalence risk group**	100% *	3.39	(3.35, 3.43)	16.07	(15.15, 16.98)				
** High prevalence groups**	85% *	3.03	(3.00, 3.06)	24.93	(24.19, 25.68)				
** Increase in price**	180% †	3.01	(2.98, 3.04)	25.53	(24.73, 26.33)				

^a^ Impact on firearm-related homicide of interventions implemented indepedentently of any other interventions.

^b^ Impact on firearm-related homicide of combined groups of interventions implemented simultaneously.

* % of efficacy needed among prevalence group.

^†^ Increase in price (% increase) needed to achieve desired reduction.

### Estimated impact of high-risk approaches and price increases on firearm-related homicide

As shown in the first row of Table 2, even if 100% of individuals in the low-prevalence prohibited group were identified and effectively disqualified from gun purchasing, the maximum reduction of firearm-related homicide would be an average of 1.96% (95% CI 1.07–2.85%).

As the size of the disqualified group increased, the proportion of the group that would have to be effectively denied to reduce firearm-related homicide by 5% decreased: 25% among a moderate prevalence prohibited group (5.15% reduction, 95% CI 3.35–5.95%), or 12% among a high prevalence prohibited group (5.33% reduction, 95% CI 4.45–6.20%). In contrast, an 18% increase in price would reduce firearm homicide by 5.37% (95% CI 4.43–6.31%).

We then increased the desired reduction in firearm-related homicide to 10% using the same approach. To reduce firearm-related homicide by ~10%, firearm disqualification would need to be 55% effective among a moderate prevalence prohibited group (9.99% reduction, 95% CI 9.37–10.62%) or 25% among a high prevalence prohibited group (10.23% reduction, 95% CI 9.39–11.08%). Alternatively, overall firearm price would need to increase by 35% (9.65% reduction, 95% CI 8.78–10.52%).

We proceeded to evaluate the percent efficacy needed for increasing reductions in homicide. When the desired reduction was more extreme, the proportion of prohibited individuals who needed to be disarmed increased to 100% regardless of prohibited group prevalence, and even then, desired reductions could not be achieved in some cases.

Reducing the firearm-related homicide rate by 30% or more could not be achieved by disqualifying any prohibited group that we modeled, even at 100% efficacy. In addition, the price of firearms would need to increase by an estimated 300% to achieve this reduction (30.54% reduction, 95% CI 29.83–31.26%).

### Estimated impact of combined high-risk and population approaches on firearm-related homicide

Table 2 also presents the additional decrease in firearm-related homicide resulting from implementing all of the high-risk approaches (at the level of effective enforcement identified for an isolated desired effect) simultaneously, along with the price increase necessary to achieve the desired result.

For example, if the percent efficacy of firearm disqualification was enforced for low, moderate, and high prevalence prohibited groups at 100%, 25%, and 12%, respectively, while simultaneously increasing price by 18%, we estimated that firearm-related homicide would decrease by 12.34% (95% CI 11.40–13.27%), as opposed to the ~5% reduction each intervention could achieve alone.

For the 5%, 10%, and 15% groups, the increase in price coupled with reductions in ownership by prohibited groups achieved approximately double the decrease in firearm-related homicide than any intervention could achieve in isolation.

### Sensitivity analysis

#### Changing the price elasticity of firearm purchases

Results are shown in S2 Appendix. Overall, the results confirm our initial analyses, where combined interventions result in approximately double the decrease in firearm-related homicide. However, to reduce firearm-related homicide, you would need to increase the price by approximately 50% with a 2% increase in price elasticity and double the increase in price with a 0.5% increase in price elasticity.

#### Reducing the efficacy of price increases through changes in illicit markets

Because it is difficult to estimate the extent to which the illicit firearm market changes following a price change in the licit market using existing data, sensitivity analyses provided insight into the potential impact of increased illicit gun purchases on firearm-related homicide reductions. We increased firearm carrying for each percentage increase in price by 5%, 10%, and 20%. The results are shown in S2 Appendix. Increasing the percentage of gun carrying even by 20% did not affect the impact on firearm-related homicide. As firearm ownership accounts for most firearms in the model (even with the most restrictive intervention, ownership prevalence is still >13%), even increasing carrying by 20% is still less than a 1% increase in total firearm availability in the model. Thus this small increase combined with the decrease in ownership does not affect the firearm-related homicide rate.

## Discussion

We simulated a hypothetical example of how restrictions on firearm access can have heterogeneous impacts on firearm-related homicide based on the size of the group restricted and whether a high-risk, population-based, or combined approach is used. Our hypothetical experiment found that modest (5–10%) reductions in firearm homicide were achieved through firearm disqualification among individuals meeting hypothetical disqualification criteria, even when those high-risk approaches were imperfectly enforced. The magnitude of the effect depends on the size of the group disqualified, the level of enforcement, and the concentration of firearm violence in each group. The same reductions could be achieved with increases in firearm price of about 18–35%. For larger decreases in firearm homicide (20% or higher), firearm disqualification in the hypothetical experiment needed to be virtually perfectly implemented and effective, and decreases of similar magnitude with increases in firearm price became unfeasible. Combined implementation of firearm purchase denial criteria for prohibited groups and price increases produced the greatest reduction in firearm-related homicide.

These results illustrated the principle that the effectiveness of a high-risk approach is dependent on the size of the groups considered to be “high risk,” and simultaneously, the strength of the relationship between the high-risk criterion and firearm violence and the extent to which the “high-risk” group is effectively disarmed. All three are critical in conjunction; that is, the size of the group is meaningless if individuals in the group do not have high concentration of the outcome, or if the intervention is ineffective. Prevalence, however, is also key. Because the prevalence of psychiatric hospitalizations in the population was low [62,63,87], and the causal effect of psychiatric illness on perpetration of firearm violence is weak and nuanced [63], disqualifying this group from purchasing firearms resulted in a small reduction in firearm-related homicide, even assuming perfect enforcement. While there may be other reasons to consider firearm control among those with particular psychiatric disorders, such as reduced suicide [88], our model suggests there would be a negligible impact on homicide.

In contrast, those convicted of a misdemeanor or felony are a much larger group, and the associations with violent perpetration are stronger [11,89], which leads to a larger reduction in firearm-related homicide even under imperfect enforcement of firearm purchase disqualifications. The high-risk approach faces numerous challenges, however. Available data from Massachussetts indicate that over 60% of individuals arrested for firearm violence perpetration are disqualified from gun ownership by federal law [90]; to the extent that these individuals owned or carried guns despite disqualification, it reduces the utility of high-risk approaches focused on ownership disqualification. Approaches to high-risk firearm owner identification and enforcement of firearm removal such as recent efforts in California provide frameworks to consider how to improve the efficacy of disqualification policies [91]. These gun-disqualification considerations, however, should also be evaluated in light of economic and racial injustices in the criminal legal system [92], as well as potential increases in stigma of criminal legal system involvement if more civil liberties are eroded with convictions.

An alternative, or adjunctive, hypothetical approach would be a population-based approach, including to increase price. While this approach burdens low-income more than high-income groups, it does not target any group in particular for a legal restriction on firearm access. We find that modest price increases can have the same impact on the population firearm homicide rate as enacting purchase denials for prohibited groups in synthetic NYC, considering that such denials are imperfectly implemented across US states. The example of price control is just one of an array of population-based strategies that countries have considered, and public health models of potential efficacy could feasibly be conducted for any number of population-based strategies; our model provides an example of one strategy, and the conclusion that we draw illustrates fundamental principles of public health. Indeed, a key takeaway of our study is that adopting high-risk versus population-based approaches should not be an “either-or” question. The greatest reduction in firearm-related homicide was achieved in this hypothetical example by adopting a combination of high-risk approaches that prohibited individuals meeting criteria for (adapted) firearm disqualification criteria, combined with population-based approaches that increased the price of firearms. Further, price increases affect many other areas of the firearm market, and are influenced by exogenous policies, laws, and events, thus price increases may have variable effectiveness and sustainability based on other factors [31,93]. Given the concerns about stigmatizing specific groups of people and enforcing firearm purchase disqualification criteria, future research should build on this work by identifying specific policies that effectively combine high-risk and population-based approaches. Additional ‘high-risk’ approaches such as extreme risk protection orders (or “red flag laws”) [94,95] and firearm buy-back programs [96] may be useful targets for estimating impacts on homicide in future work.

The present simulation study is a hypothetical example that does not perfectly reflect reality; for example, there are other high-risk groups that we could have modeled (e.g., those under 21), gun disqualifications differ at local and state levels, and further, the vast majority (over 60%) of people who perpetrate homicide already have a disqualification from gun ownership [90]. Further, we made broad assumptions about gun purchase elasticity that may not reflect the number of guns owned by consumers, specific products, differences in elasticity based on legal versus illegal ownership/carrying, or the purchase patterns of guns at different price points. The purpose of our simulation was to illustrate the underlying principles of the high-risk and population-based approaches using hypothetical examples, but not to recreate true underlying dynamics of disqualification and purchasing behavior. Further, modeling firearm ownership and firearm carrying remain challenging due to the lack of comprehensive data.

While there is tremendous interest in reducing the impact of gun violence in the United States, there remains substantial disagreement about how to achieve meaningful and sustained reductions in violence using a policy response. Price increases have been a cornerstone of public health responses for many other products that damage the public’s health, and modest increases in price can have even more impact when coupled with high-risk interventions that reduce firearm access. As always, however, equity and civil liberty concerns need to be constantly evaluated; too often policy responses in the US have the unintended consequence of increasing health and criminal justice inequities, and as these decisions are increasingly debated in public and private discourse, continued attention to reducing inequity should be at the forefront.

## Supporting information

S1 Appendix Description of ABM using the ODD protocol. Fig A1.1. Diagram of relations between agent, social network, and neighborhood characteristics in the agent-based model. Table A1.1. Agent, social network, and neighborhood parameters, values, data sources, and update rules. Table A1.2. Agent-based model initialization parameters and default values. Fig A1.2. Flow diagram illustrating steps in model initialization. Fig A1.3. Flow diagram illustrating processes occurring at each step of the model.
(DOCX)

S2 Appendix Sensitivity analyses. Table A2.1. Simulation of gun-related homicide in New York City after implementing firearm disqualification and price increases with a 1% price elasticity, increase in carrying among those who do not own by 5%. Table A2.2. Simulation of gun-related homicide in New York City after implementing firearm disqualification and price increases with a 1% price elasticity, increase in carrying among those who do not own by 10%. Table A2.3. Simulation of gun-related homicide in New York City after implementing firearm disqualification and price increases with a 1% price elasticity, increase in carrying among those who do not own by 20%.
(DOCX)
