# Supplementary material for: Simulating the bounds of plausibility: Estimating the impact of high-risk versus population-based approaches to prevent firearm injury
Source: PLoS One. 2022 Jun 2;17(6):e0269372. doi: 10.1371/journal.pone.0269372 (PMC9162316; doi:10.1371/journal.pone.0269372)
Supplement: S2 Appendix — Table A2.1. Simulation of gun-related homicide in New York City after implementing firearm disqualification and price increases with a 1% price elasticity, increase in carrying among those who do not own by 5%. Table A2.2. Simulation of gun-related homicide in New York City after implementing firearm disqualification and price increases with a 1% price elasticity, increase in carrying among those who do not own by 10%. Table A2.3. Simulation of gun-related homicide in New York City after implementing firearm disqualification and price increases with a 1% price elasticity, increase in carrying among those who do not own by 20%. (DOCX) [file pone.0269372.s002.docx]

**Appendix 2. Sensitivity analyses**

Table A2.1. Simulation of gun-related homicide in New York City after implementing firearm disqualification and price increases with a 1% price elasticity, increase in carrying among those who do not own by 5%.

| **Intervention** | **Percent Efficacy** | **Individual Intervention  Effects** | | | | **Combined Intervention  Effects** | | | |
| --- | --- | --- | --- | --- | --- | --- | --- | --- | --- |
|  |  | **Firearm-Related  Homicide Rate/100,000** | | **% Decrease in Firearm- Related Homicide** | | **Firearm-Related  Homicide Rate/100,000** | | **% Decrease in Firearm- Related Homicide** | |
|  |  | **Mean** | **(95% CI)** | **Mean** | **(95% CI)** | **Mean** | **(95% CI)** | **Mean** | **(95% CI)** |
| **Baseline** |  | 4.04 | (4.00, 4.08) |  |  | 4.04 | (4.00, 4.08) |  |  |
| **Target: 5% Decrease in Homicide** | | | | | | | | | |
| **Low prevalence groups** | 100% * | 3.96 | (3.92, 4.00) | 1.96 | (1.07, 2.85) | 3.49 | (3.46, 3.52) | 13.61 | (12.97, 14.25) |
| **Medium prevalence group** | 25% * | 3.83 | (3.80, 3.86) | 5.15 | (4.35, 5.95) |  |  |  |  |
| **High prevalence groups** | 12% * | 3.82 | (3.79, 3.86) | 5.33 | (4.45, 6.20) |  |  |  |  |
| **Increase in price** | 18% † | 3.82 | (3.78, 3.86) | 5.46 | (4.52, 6.41) |  |  |  |  |
| **Target: 10% Decrease in Homicide** | | | | | | | | | |
| **Low prevalence groups** | 100% * | 3.96 | (3.92, 4.00) | 1.96 | (1.07, 2.85) | 3.19 | (3.16, 3.22) | 21.02 | (20.18, 21.86) |
| **Medium prevalence group** | 55% * | 3.64 | (3.61, 3.66) | 9.99 | (9.37, 10.62) |  |  |  |  |
| **High prevalence groups** | 25% * | 3.63 | (3.59, 3.66) | 10.23 | (9.39, 11.08) |  |  |  |  |
| **Increase in price** | 35% † | 3.63 | (3.6, 3.66) | 10.10 | (9.32, 10.88) |  |  |  |  |
| **Target: 15% Decrease in Homicide** | | | | | | | | | |
| **Low prevalence groups** | 100% * | 3.96 | (3.92, 4.00) | 1.96 | (1.07, 2.85) | 2.82 | (2.78, 2.85) | 30.24 | (29.35, 31.14) |
| **Medium prevalence group** | 100% * | 3.39 | (3.35, 3.43) | 16.07 | (15.15, 16.98) |  |  |  |  |
| **High prevalence groups** | 40% * | 3.42 | (3.39, 3.45) | 15.29 | (14.48, 16.11) |  |  |  |  |
| **Increase in price** | 70% † | 3.41 | (3.38, 3.45) | 15.49 | (14.63, 16.36) |  |  |  |  |
| **Target: 20% Decrease in Homicide** | | | | | | | | | |
| **Low prevalence groups** | 100% * | 3.96 | (3.92, 4.00) | 1.96 | (1.07, 2.85) | 2.65 | (2.62, 2.68) | 34.47 | (33.76, 35.17) |
| **Medium prevalence group** | 100% * | 3.39 | (3.35, 3.43) | 16.07 | (15.15, 16.98) |  |  |  |  |
| **High prevalence groups** | 60% * | 3.21 | (3.18, 3.24) | 20.55 | (19.78, 21.32) |  |  |  |  |
| **Increase in price** | 110% † | 3.20 | (3.16, 3.24) | 20.68 | (19.7, 21.66) |  |  |  |  |
| **Target: 25% Decrease in Homicide** | | | | | | | | | |
| **Low prevalence groups** | 100% * | 3.96 | (3.92, 4.00) | 1.96 | (1.07, 2.85) | 2.47 | (2.44, 2.49) | 38.97 | (38.28, 39.65) |
| **Medium prevalence group** | 100% * | 3.39 | (3.35, 3.43) | 16.07 | (15.15, 16.98) |  |  |  |  |
| **High prevalence groups** | 85% * | 3.03 | (3.00, 3.06) | 24.93 | (24.19, 25.68) |  |  |  |  |
| **Increase in price** | 180% † | 2.99 | (2.95, 3.02) | 26.07 | (25.16, 26.98) |  |  |  |  |
| ^a^ Impact on firearm-related homicide of interventions implemented independently of any other interventions | | | | | | | | | |
| ^b^ Impact on firearm-related homicide of combined groups of interventions implemented simultaneously | | | | | | | | | |
| ^*^ % of efficacy needed among prevalence group | | | | | | | | | |
| ^†^ Increase in price (% increase) needed to achieve desired reduction | | | | | | | | | |

| **Intervention** | **Percent Efficacy** | **Individual Intervention  Effects** | | | | **Combined Intervention  Effects** | | | |
| --- | --- | --- | --- | --- | --- | --- | --- | --- | --- |
|  |  | **Firearm-Related  Homicide Rate/100,000** | | **% Decrease in Firearm- Related Homicide** | | **Firearm-Related  Homicide Rate/100,000** | | **% Decrease in Firearm- Related Homicide** | |
|  |  | **Mean** | **(95% CI)** | **Mean** | **(95% CI)** | **Mean** | **(95% CI)** | **Mean** | **(95% CI)** |
| **Baseline** |  | 4.04 | (4.00, 4.08) |  |  | 4.04 | (4.00, 4.08) |  |  |
| **Target: 5% Decrease in Homicide** | | | | | | | | | |
| **Low prevalence groups** | 100% * | 3.96 | (3.92, 4.00) | 1.96 | (1.07, 2.85) | 3.54 | (3.5, 3.57) | 12.44 | (11.62, 13.27) |
| **Medium prevalence group** | 25% * | 3.83 | (3.80, 3.86) | 5.15 | (4.35, 5.95) |  |  |  |  |
| **High prevalence groups** | 12% * | 3.82 | (3.79, 3.86) | 5.33 | (4.45, 6.20) |  |  |  |  |
| **Increase in price** | 18% † | 3.85 | (3.81, 3.89) | 4.72 | (3.72, 5.73) |  |  |  |  |
| **Target: 10% Decrease in Homicide** | | | | | | | | | |
| **Low prevalence groups** | 100% * | 3.96 | (3.92, 4.00) | 1.96 | (1.07, 2.85) | 3.19 | (3.16, 3.23) | 20.91 | (20.08, 21.74) |
| **Medium prevalence group** | 55% * | 3.64 | (3.61, 3.66) | 9.99 | (9.37, 10.62) |  |  |  |  |
| **High prevalence groups** | 25% * | 3.63 | (3.59, 3.66) | 10.23 | (9.39, 11.08) |  |  |  |  |
| **Increase in price** | 35% † | 3.64 | (3.61, 3.68) | 9.78 | (8.98, 10.57) |  |  |  |  |
| **Target: 15% Decrease in Homicide** | | | | | | | | | |
| **Low prevalence groups** | 100% * | 3.96 | (3.92, 4.00) | 1.96 | (1.07, 2.85) | 2.83 | (2.8, 2.86) | 29.87 | (29.15, 30.6) |
| **Medium prevalence group** | 100% * | 3.39 | (3.35, 3.43) | 16.07 | (15.15, 16.98) |  |  |  |  |
| **High prevalence groups** | 40% * | 3.42 | (3.39, 3.45) | 15.29 | (14.48, 16.11) |  |  |  |  |
| **Increase in price** | 70% † | 3.39 | (3.35, 3.42) | 16.10 | (15.23, 16.96) |  |  |  |  |
| **Target: 20% Decrease in Homicide** | | | | | | | | | |
| **Low prevalence groups** | 100% * | 3.96 | (3.92, 4.00) | 1.96 | (1.07, 2.85) | 2.65 | (2.62, 2.67) | 34.51 | (33.88, 35.14) |
| **Medium prevalence group** | 100% * | 3.39 | (3.35, 3.43) | 16.07 | (15.15, 16.98) |  |  |  |  |
| **High prevalence groups** | 60% * | 3.21 | (3.18, 3.24) | 20.55 | (19.78, 21.32) |  |  |  |  |
| **Increase in price** | 110% † | 3.22 | (3.19, 3.25) | 20.21 | (19.49, 20.93) |  |  |  |  |
| **Target: 25% Decrease in Homicide** | | | | | | | | | |
| **Low prevalence groups** | 100% * | 3.96 | (3.92, 4.00) | 1.96 | (1.07, 2.85) | 2.45 | (2.43, 2.48) | 39.23 | (38.52, 39.93) |
| **Medium prevalence group** | 100% * | 3.39 | (3.35, 3.43) | 16.07 | (15.15, 16.98) |  |  |  |  |
| **High prevalence groups** | 85% * | 3.03 | (3.00, 3.06) | 24.93 | (24.19, 25.68) |  |  |  |  |
| **Increase in price** | 180% † | 2.96 | (2.93, 3) | 26.60 | (25.78, 27.42) |  |  |  |  |
| ^a^ Impact on firearm-related homicide of interventions implemented independently of any other interventions | | | | | | | | | |
| ^b^ Impact on firearm-related homicide of combined groups of interventions implemented simultaneously | | | | | | | | | |
| ^*^ % of efficacy needed among prevalence group | | | | | | | | | |
| ^†^ Increase in price (% increase) needed to achieve desired reduction | | | | | | | | | |

Table A2.2. Simulation of gun-related homicide in New York City after implementing firearm disqualification and price increases with a 1% price elasticity, increase in carrying among those who do not own by 10%.

Table A2.3. Simulation of gun-related homicide in New York City after implementing firearm disqualification and price increases with a 1% price elasticity, increase in carrying among those who do not own by 20%.

| **Intervention** | **Percent Efficacy** | **Individual Intervention  Effects** | | | | **Combined Intervention  Effects** | | | |
| --- | --- | --- | --- | --- | --- | --- | --- | --- | --- |
|  |  | **Firearm-Related  Homicide Rate/100,000** | | **% Decrease in Firearm- Related Homicide** | | **Firearm-Related  Homicide Rate/100,000** | | **% Decrease in Firearm- Related Homicide** | |
|  |  | **Mean** | **(95% CI)** | **Mean** | **(95% CI)** | **Mean** | **(95% CI)** | **Mean** | **(95% CI)** |
| **Baseline** |  | 4.04 | (4.00, 4.08) |  |  | 4.04 | (4.00, 4.08) |  |  |
| **Target: 5% Decrease in Homicide** | | | | | | | | | |
| **Low prevalence groups** | 100% * | 3.96 | (3.92, 4.00) | 1.96 | (1.07, 2.85) | 3.52 | (3.49, 3.56) | 12.76 | (11.91, 13.61) |
| **Medium prevalence group** | 25% * | 3.83 | (3.80, 3.86) | 5.15 | (4.35, 5.95) |  |  |  |  |
| **High prevalence groups** | 12% * | 3.82 | (3.79, 3.86) | 5.33 | (4.45, 6.20) |  |  |  |  |
| **Increase in price** | 18% † | 3.83 | (3.79, 3.87) | 5.14 | (4.17, 6.12) |  |  |  |  |
| **Target: 10% Decrease in Homicide** | | | | | | | | | |
| **Low prevalence groups** | 100% * | 3.96 | (3.92, 4.00) | 1.96 | (1.07, 2.85) | 3.20 | (3.16, 3.24) | 20.81 | (19.91, 21.7) |
| **Medium prevalence group** | 55% * | 3.64 | (3.61, 3.66) | 9.99 | (9.37, 10.62) |  |  |  |  |
| **High prevalence groups** | 25% * | 3.63 | (3.59, 3.66) | 10.23 | (9.39, 11.08) |  |  |  |  |
| **Increase in price** | 35% † | 3.65 | (3.62, 3.69) | 9.56 | (8.75, 10.37) |  |  |  |  |
| **Target: 15% Decrease in Homicide** | | | | | | | | | |
| **Low prevalence groups** | 100% * | 3.96 | (3.92, 4.00) | 1.96 | (1.07, 2.85) | 2.82 | (2.79, 2.84) | 30.29 | (29.74, 30.84) |
| **Medium prevalence group** | 100% * | 3.39 | (3.35, 3.43) | 16.07 | (15.15, 16.98) |  |  |  |  |
| **High prevalence groups** | 40% * | 3.42 | (3.39, 3.45) | 15.29 | (14.48, 16.11) |  |  |  |  |
| **Increase in price** | 70% † | 3.42 | (3.38, 3.45) | 15.45 | (14.66, 16.24) |  |  |  |  |
| **Target: 20% Decrease in Homicide** | | | | | | | | | |
| **Low prevalence groups** | 100% * | 3.96 | (3.92, 4.00) | 1.96 | (1.07, 2.85) | 2.63 | (2.6, 2.66) | 34.86 | (34.09, 35.63) |
| **Medium prevalence group** | 100% * | 3.39 | (3.35, 3.43) | 16.07 | (15.15, 16.98) |  |  |  |  |
| **High prevalence groups** | 60% * | 3.21 | (3.18, 3.24) | 20.55 | (19.78, 21.32) |  |  |  |  |
| **Increase in price** | 110% † | 3.24 | (3.21, 3.27) | 19.72 | (18.93, 20.51) |  |  |  |  |
| **Target: 25% Decrease in Homicide** | | | | | | | | | |
| **Low prevalence groups** | 100% * | 3.96 | (3.92, 4.00) | 1.96 | (1.07, 2.85) | 2.47 | (2.44, 2.5) | 38.83 | (38.08, 39.58) |
| **Medium prevalence group** | 100% * | 3.39 | (3.35, 3.43) | 16.07 | (15.15, 16.98) |  |  |  |  |
| **High prevalence groups** | 85% * | 3.03 | (3.00, 3.06) | 24.93 | (24.19, 25.68) |  |  |  |  |
| **Increase in price** | 180% † | 3.01 | (2.98, 3.05) | 25.39 | (24.61, 26.17) |  |  |  |  |
| ^a^ Impact on firearm-related homicide of interventions implemented independently of any other interventions | | | | | | | | | |
| ^b^ Impact on firearm-related homicide of combined groups of interventions implemented simultaneously | | | | | | | | | |
| ^*^ % of efficacy needed among prevalence group | | | | | | | | | |
| ^†^ Increase in price (% increase) needed to achieve desired reduction | | | | | | | | | |
